# Supplementary material for: H2A histone-fold and DNA elements in nucleosome activate SWR1-mediated H2A.Z replacement in budding yeast
Source: eLife. 2015 Jun 27;4:e06845. doi: 10.7554/eLife.06845 (PMC4508883; doi:10.7554/eLife.06845)
Supplement: Supplementary file 1. — Genotype of strains used in this study, related to Figure 1. DOI: http://dx.doi.org/10.7554/eLife.06845.013 [file elife06845s001.docx]

## Supplementary File 1. Genotype of strains used in this study, related to Fig. 1

| STRAIN | GENOTYPE | SOURCE |
| --- | --- | --- |
| SWR1-Flag htz1∆ | W1588C-4C swr1::SWR1-3Flag-P-KanMX-P htz1::natMX4 | Kind gift from Wei-Hua Wu |
| FY406 | MATa (*hta1-htb1*)∆::*LEU2*, (*hta2 htb2*)∆::*TRP1*, *his3∆200 leu2∆1 ura3-52 trp1∆63 lys2-128∆* <pSAB6 (*HTA1-HTB1-URA3*) | Winston lab |
| FY406 HTZ1-HA | FY406, HTZ1-HA:KanMx | This work |
| H2A WT | MATa (hta1-htb1)∆::LEU2, (*hta2* htb2)∆::TRP1, his3∆200 leu2∆1 ura3-52 trp1∆63 lys2-128∆ HTZ1-HA:KanMx <pZS66 (HTA1-HTB1-HIS3) | This work |
| H2A  G47K | MATa (hta1-htb1)∆::LEU2, (*hta2* htb2)∆::TRP1, his3∆200 leu2∆1 ura3-52 trp1∆63 lys2-128∆ HTZ1-HA:KanMx  <pZS66 (hta1[G47K]-HTB1-HIS3) | This work |
| H2A  P49A | MATa (hta1-htb1)∆::LEU2, (*hta2* htb2)∆::TRP1, his3∆200 leu2∆1 ura3-52 trp1∆63 lys2-128∆ HTZ1-HA:KanMx  <pZS66 (hta1[P49A]-HTB1-HIS3) | This work |
| H2A  G47K, P49A | MATa (hta1-htb1)∆::LEU2, (*hta2* htb2)∆::TRP1, his3∆200 leu2∆1 ura3-52 trp1∆63 lys2-128∆ HTZ1-HA:KanMx  <pZS66 (hta1[G47K, P49A]-HTB1-HIS3) | This work |
| H2A WT, H2A-M4 | MATa (*hta1-htb1*)∆::*LEU2*, (*hta2 htb2*)∆::*TRP1*, *his3∆200 leu2∆1 ura3-52 trp1∆63 lys2-128∆* HTZ1-HA:KanMx  <pSAB6 (*HTA1-HTB1-URA3*) <pZS66 (hta1[M4]-HTB1-HIS3) | This work |
